# Supplementary material for: ENTRAIN: integrating trajectory inference and gene regulatory networks with spatial data to co-localize the receptor–ligand interactions that specify cell fate
Source: Bioinformatics. 2023 Dec 19;39(12):btad765. doi: 10.1093/bioinformatics/btad765 (PMC10752580; doi:10.1093/bioinformatics/btad765)
Supplement: btad765_Supplementary_Data [file btad765_supplementary_data.zip › Kyaw et al_Supplementary_Information_FINAL_231213.docx]

**SUPPLEMENTARY METHODS**

**Extracting regulatory information from NicheNet**

Expression dynamics during differentiation are likely to be a manifestation of cell-intrinsic and cell-extrinsic regulatory programmes. To demarcate these two factors, the algorithm’s second step unites prior knowledge of ligand-receptor pairs and their corresponding intracellular regulatory interactions to determine potential ligands driving the observed TRAINing gene expression dynamics.

Under the assumption that the microenvironmental niche has a quantifiable contribution to gene expression dynamics in differentiation, we require a database that predicts which target genes are subject to regulation by ligand-receptor pairs. ENTRAIN extracts this information from NicheNet1, which unites traditional ligand-receptor signalling to downstream transcriptional regulation.

NicheNet generates ligand-target regulatory potentials by combining publicly available GRN databases (including intra- and extra-cellular signaling) with microarray data comprising RNA expression profiles from 111 *in vitro* ligand treatment experiments. It computes a weighted sum of 14 regulatory network databases and 13 signaling network databases. Weights were calculated by multi-objective parameter optimization such that the weights maximized the NicheNet model’s ability to classify differentially expressed genes upon *in vitro* ligand treatment.

At this point, we have a network between extracellular ligands and genes (n x m), capturing the LR binding and activation of downstream regulators, and a second intracellular network between genes and other genes (m x m), a subset of which contains the interaction between regulators and target genes. While the n x m matrix represents a ligand-gene regulatory potential, this network was considered naïve because it only considered direct links between ligands and genes. The Personalized PageRank (PPR) algorithm2 was used to update the naïve (n x m) matrix with indirect interactions (e.g., regulatory signal that is propagated through layers of intermediary regulators). This calculates, for each ligand, the distance of a given regulator from that ligand, taking account the network edge weights optimized in the previous step. The resulting ligand-gene weighted matrix was then matrix multiplied with the (m x m) matrix to generate the final ligand-gene matrix, denoted as the ‘regulatory potential’ of the interaction.

Because the PPR is calculated on a ligand-by-ligand approach, the ligand regulatory potentials are relative and not comparable across different ligands, only within the same ligand. In this methodology, both the quality of the data sources (e.g., multiple data sources corroborating the same interaction), as well as the ability of the regulatory potential scores to predict DEGs in an *in vitro* ligand treatment dataset and inferred indirect interactions (via the PPR algorithm), contribute towards the calculation of the final regulatory potentials.

We first identified active LR pairs amongst the trajectory cells (‘receivers’) and the remaining cells in the dataset (‘senders’), using NicheNet as prior knowledge of possible ligand-receptor interactions. With the assumption that high LR expression levels do not necessarily correlate to significance in driving differentiation trajectories, we determined LR pairs for further analysis if they fulfilled two criteria: 1. They are expressed by a sufficient proportion of cells in the dataset (default >0 counts in at least 10% of cells). 2. The corresponding receptors are expressed by a sufficient proportion of differentiating cells (default >0 counts in at least 10% of differentiating cells). Of the ligands that meet the criteria, we extracted their respective downstream target regulation scores from the NicheNet database. These are vectors representing the ability of a given ligand to regulate every human gene. Thus, each ligand is associated with a vector of length *g*, where *g* is the number of human genes in the database, and each element of the vector is a number (a “regulatory potential”) representing the strength of the regulatory relationship between the ligand and a given gene.

**Calculation of cell-wise influences**

Differentiating cells exhibit changes in receptor expression and regulatory wiring as they progress along a developmental process. Because of this, we hypothesized that certain stages of a developmental process will be more influenced by environmental signalling than other stages. We thus wished to produce a more granular, cell-wise measure of ligand influence that encapsulates this behaviour. To do this we calculated pseudotime-expression covariances along a rolling window of cells along pseudotime, restricted to separate branches (**Supplementary** **Algorithm 1**). We used a default window size *w* and step size *s* of 10% and 2% of the cells in the trajectory branch, respectively. This ‘local covariance’ quantifies a gene’s expression dynamics within a rolling window of differentiating cells. To this end, we fit a second round of random forest models to each rolling window, such that every branch is now subject to an additional 50 ‘local’ model fits corresponding to 50 rolling windows along the branch. The number of local model fits is dependent on the values of *s* and ; 50 rolling windows is the behaviour when *s* and are assigned default values. We used regulatory potentials from the top 5 ligands as the predictor variable (a *x* 5 matrix with default parameters) and the local covariances as the response variable. For step sizes greater than 1, we linearly interpolate values for cells which are skipped.



Resultant %V.E.values denote the confidence of the NicheNet fit at each of the 50 windows. Genes possessing high covariance with pseudotime are assumed to be important for trajectory determination, and we are interested in the subset of those that are under environmental control. Some of these high-covariance genes will not be under extracellular control and consequently exhibit a low %V.E. value when fitted to NicheNet. On the other hand, high covariance genes that are also under extracellular control will exhibit both high covariances and a confident fit (increased %V.E.) to NicheNet. As a result, these window %V.E. values can be interpreted as the degree of environmental dependence across different stages of the trajectory. Ultimately, every trajectory branch is subject to one ‘branch-wide’ model fit that determines the top few ligands of interest, and 50 ‘local’ model fits that assess where their regulatory effects are most noticeable. Cells with cell-intrinsic drivers would be expected to exhibit low, negative, or widely varying %V.E. values as the model cannot accurately fit environmental regulators to the observed expression dynamics in that window, while the opposite is true for highly environmentally dependent windows. We note that the term ‘cell-wise’ is slightly misleading, as the observed expression dynamics are deduced from the covariances of many neighbouring cells in a rolling window of observations rather than a single cell.

**Supplementary** **Algorithm 1:**

**Inputs**

Trajectory branches: **b**, a collection of ( x ) cell by gene matrices bi, one for each branch (pre-dimension reduction). Each cell is assigned a pseudotime

NicheNet Ligand Target Database**:** ( x )matrix

: step size, an integer denoting the interval (in # of cells) between successive rolling windows.

: window width, an integer denoting the size (in # of cells) of the rolling window.

**For each** branch bi in **b**

Sort cells in b*i* by pseudotime

**for** ,

Select a window of cells , where , and denotes the window centre.

Calculate covariances between pseudotimes and gene expression in the window. Specifically,

, where denotes the gene expression of gene in cell

Fit to with randomForest

Store value from randomForest for the window centre cell in a vector

**if** ()

Linearly interpolate values of for all cells that were not assigned as a window centre. For example, If = 5, every 5th cell will be denoted a window centre, meaning that 4 out of every 5 cells in the dataset will be missing an value. These 4 cells will have their values interpolated.

**Supplementary** **Algorithm 2:**

**Inputs**

**:** Velocity matrix, a ( x ) cell by gene matrix denoting RNA velocities.

**:** NicheNet Ligand Target Database( x matrix, where is the number of active ligands present in the cell population

**P:** Leiden clustering resolution parameter (0 1)

1. Cluster using Leiden algorithm with resolution , resulting in clusters and a corresponding number of matrices of shape x , where is the number of cells in a given cluster

**For each**  in **:**

1. Calculate gene likelihoodsusing scvelo.tl.recover_dynamics() on **,** the corresponding velocity matrix for cells in cluster . This generates a likelihoods vector of length , where , containing likelihoods calculated for genes denoted as velocity genes.
2. Define **.** In other words,consists of rows from that correspond to genes with calculated fit likelihoods.
3. Fit to with randomForest.
4. Append (% velocity variance explained by ligands for cluster ) and (vector of length , denoting variable importance) to vectors and .

**return:** ,

**Benchmarking and Validation**

To select the clusters to benchmark against, we conducted a velocity confidence analysis using scVelo to identify the most reliable velocities for comparison. In both the Manno and Ratz datasets, velocity clusters corresponding to neuroblasts/neurons and oligodendrocytes, respectively, exhibited the highest confidence among clusters with positive variance explained (**Supplementary Figure S2**). As a result, our benchmarking efforts focused on these specific velocity clusters for comparison against both the literature and other methods.

**Benchmarking against NicheNet**

NicheNet was applied to the BME/EN datasets using default parameters. Sender cells were set as ‘undefined’ to determine LR pairs in an agnostic manner. “Condition_oi” (condition of interest) and “condition_reference” were defined as Pre-B Cells and Progenitor cells (for Pre-B cell differentiation), or “Neuron” and “Neuroblast” (for Neuroblast differentiation). NicheNet results were then ranked by Pearson correlation to determine the ranking order.

**Benchmarking against CellCall**

CellCall was applied to the BME/EN datasets using default parameters provided in documentation. CellCall results were then filtered to ligand-receptor interactions involving the cell type of interest (i.e., Pre-B cells or neuroblasts). Ligands were then ranked by scores to determine the ranking order.

**Literature Curation**

Because NicheNet and ENTRAIN return a scored list of all active ligands, without defining an ‘active ligand’, we compared the top 10 ligands from each method for literature curation.

For each ligand, a literature search was performed following these specifications:

1. The ligand has been mentioned to be involved in extracellular signalling
2. The ligand has been reported to be involved in differentiation or development of the cell type of interest: Pre-B cell development, or neurogenesis, or oligodendrocyte differentiation.
3. Ligands reported to be involved regulation in the cell type of interest but not in differentiation (e.g., lymphoblastic lymphoma, glioblastoma multiforme), or involved in non-developmental differentiation (e.g., post-germinal centre B cell differentiation) were excluded.

**Validation Metrics and Simulated Data**

**Simulated Data with CellOracle**

We utilized CellOracle3, an *in silico* machine learning approach to simulate the effects of knocking out critical TFs on downstream GRNs. Given that some TFs are under strict environmental control via LR pairs, whilst some TFs are not, we surmised that simulating knockout of TFs under strict environmental control would be a suitable alternative to generate synthetic datasets that reconstruct the regulatory effects of LR pair binding. We hypothesized that running ENTRAIN on such datasets would recover those LR pairs known to be controlling those TFs.

For this purpose, we focussed on the TFs *Hes1* and *Hes5*, which were strongly linked to a LR pair whilst occurring in the top 15 ranked TFs by eigenvalue centrality (a measure, produced by CellOracle, which signifies TFs with significant connectivity in the gene regulatory network of the analyzed cell population). Using the Manno et al. dataset, we performed *in silico* knockouts on *Hes1* and *Hes5*, generating a 'delta' matrix ("oracle velocities") of predicted gene expression changes (**Supplementary Figure S5**). ENTRAIN analysis of these velocities identified Notch ligands Dll1, Dll3, and Jag2 within the top five ligands for both *Hes1* and *Hes5*. These findings align with known GRNs and demonstrate ENTRAIN’s compatibility and reliability with simulated datasets, highlighting its utility in exploring complex GRNs. We next cross referenced the recovered ligands with existing literature by searching literature for confirmed regulatory interactions between the ligands and Notch, Hes1, or Hes5 signaling **(Supplementary Table S3)**. If the ligand was found to have a confirmed interaction, we classified this as a true positive and visualized true/false positives using R package pROC. The resultant AUROC analysis revealed strong support, yielding scores of 0.869 and 0.822 for Hes1 and Hes5 knockout simulations, respectively.

**Subsampling**

We have subsampled the Manno et al. dataset to between 30-90% of the original cell count. We then tested how the variance explained (V.E.) was influenced by varying the subsampling fraction **(Supplementary Figure S6A-S6C).** We then evaluated the ability of ENTRAIN, when run on subsampled data, to reproduce the results of the original, non-subsampled data. We used two metrics to evaluate this, AUC and Spearman correlation, in both cases treating the non-subsampled result as ground truth**.**

**Gene Permutation Test**

We have performed gene shuffling by randomly permuting the velocity likelihoods vector (the response variable). This was used to answer two objectives:

- 1. **Calculating the likelihood of identifying a given TRAINing gene, under a null scenario (Supplementary Figure S6E)**

The permutation test for objective (a) was performed as follows:

- - 1. Generate the top ranked velocity genes on the real (non-randomized) data, using the Manno et al. neuroblast-neuron cluster as the dataset.
    2. Permutation test: Permute the gene labels.
    3. Recalculate the ranks on the permuted data.
    4. (ii) and (iii) were repeated 1000 times
    5. In the 1000 tests, we count the number of times (m) a gene ranks higher in the permuted vector than in the non-permuted vector.
    6. The false discovery rate is then .
    7. Steps (ii) to (vi) were repeated for 10 rounds to obtain 10 FDR scores per datapoint.
  1. **Computing a false discovery rate (FDR) by comparing V.E. from the original data with the V.E. obtained from permuted versions of the response variable (Supplementary Figure S6F).** This was performedwhilst varying the ‘n_top_genes’ parameter (number of TRAINing genes used). This has the side effect of determining a suitable value for the number of TRAINing genes used

The permutation test for objective (b) was performed as follows, for each value of n_top_genes (200 <= n_top_genes <= 3000, 9 values tested in total):

- - 1. Calculate the V.E. (VEorig) for the original, non-permuted data, using the n_top_genes TRAINing genes.
    2. Permute the TRAINing genes randomly, and run ENTRAIN using that vector as the response variable. Record the resulting V.E. (‘VEpermute’) and record if a false discovery is made i.e., if ‘VEpermute > VEorig.
    3. Repeat (ii) n=50 times, each time counting false discoveries, and calculate a false discovery rate by
    4. Repeat steps (i) to (iii) m = 10 times for each value of *n_top_genes*, for a total of 500 fits for the current value of *n_top_genes*.
    5. This produces a grand total of 9*m*n = 4500 tests across all values and trials. The 10 FDR measurements for each *n_top_genes* were then averaged and plotted in Supplementary Figure S6F.

**EXPERIMENTAL DETAILS**

**Mice**

6-8 week-old immunocompetent wildtype C57BL6/J mice were used. Animal experiments were approved by the Garvan Institute of Medical Research Animal Ethics Committee (ARA16/01 and ARA19/09).

**Isolation of endosteal and marrow cells**

To obtain bone and bone marrow stroma cells for scRNA-seq, mice were sacrificed via CO2 asphyxia. Femurs were harvested and soft tissue was removed. The femurs were then separated into diaphysis and metaphysis (epiphysis was removed). Marrow cells were collected by flushing the diaphysis with PBS. Endosteal cells were isolated from marrow-depleted diaphyseal and metaphyseal bone fractions by gently crushing and cutting bones and digested in 2 mg/ml of collagenase A and 2.5mg/ml of trypsin for 30 mins at 37ºC. After digestion, bone fractions were vortexed for 10s and the supernatant containing digested cells was filtered through a 100μm filter into collection tubes containing 10% FCS. Marrow cells and endosteal cells were then centrifuged at 400x g for 5 mins and resuspended in 200uL and were then stained in PBS supplemented with 2% FCS for FACS sorting.

**FACS enrichment of endosteal and marrow cells**

Cells were stained for Ter119-PE at 4ºC for 30mins and rinsed with PBS supplemented with 2% FCS. Dead cells and debris were excluded by FSC, SSC and DAPI (ThermoFisher Scientific). Cells that were viable (DAPI-negative) and negative for erythroid marker (Ter119) were sorted into PBS supplemented with 2% FCS.

**Single cell RNA-seq**

Single cells were encapsulated into emulsion droplets using the 10x Chromium (10x Genomics). scRNA-seq libraries were constructed using Chromium Single Cell 30 v2 Reagent Kit according to the manufacturer’s protocol. Briefly, FACS sorted sample volume was decreased and cells were examined under a microscope and counted with a cell counter (Thermo Fisher Scientific). Cells were then loaded in each channel with a target output of 10,000 cells. Reverse transcription and library preparation were performed on C1000 Touch Thermal cycler with 96-Deep Well Reaction Module (Bio-Rad). Amplified cDNA and final libraries were evaluated on a Agilent BioAnalyzer using a High Sensitivity DNA Kit (Agilent Technologies). Individual libraries were diluted to 4nM and pooled for sequencing. Pools were sequenced with 75 cycle run kits (26bp Read1, 8bp Index1 and 55bp Read2) on the Novaseq Sequencing System (Illumina) to 80%-90% saturation level.

**Pre-processing of 10x scRNA-seq data**

Raw sequencing data were processed using the CellRanger pipeline (10x Genomics). Count matrices were loaded into R and further processed using Seurat4. We removed all cells with fewer than 300 distinct genes observed or cells with more than 10% of unique molecular identifiers stemming from mitochondrial genes.

**Dimensionality reduction**

Dimensionality reduction was performed using gene expression data for the top 3000 variable genes. The variable genes were selected based on dispersion of binned variance to mean expression ratios using FindVariableGenes function of Seurat package4. Next, principal component analysis (PCA) was performed and the first 40 principal components were included for subsequent clustering and UMAP analysis based on manual inspection of a principal component variance plot (‘PC elbow plot’).

**Clustering and sub-clustering**

Graph-based clustering of the PCA reduced data with the Louvain Method was performed5 after computing a shared nearest neighbor graph6. The clusters were visualized on a 2D map produced with Uniform Manifold Approximation and Projection (UMAP)7. For sub-clustering, we applied the same procedure of finding variable genes, dimensionality reduction, and clustering to the restricted set of data (usually restricted to cell clusters of the same lineage).

**Differential expression of gene signatures**

The marker genes for each cluster were identified using the FindAllMarkers function and ROC-based test statistics of Seurat.

**Filtering out doublets**

It is to be expected that a fraction of data should consist of cell doublets (and to an even lesser extent of higher order multiplets) due to co-encapsulation into droplets and/or pairs of cells that were not dissociated in sample preparation. Therefore, clusters of cells expressing markers of different lineages were removed from further analysis.

**SUPPLEMENTARY FIGURES**


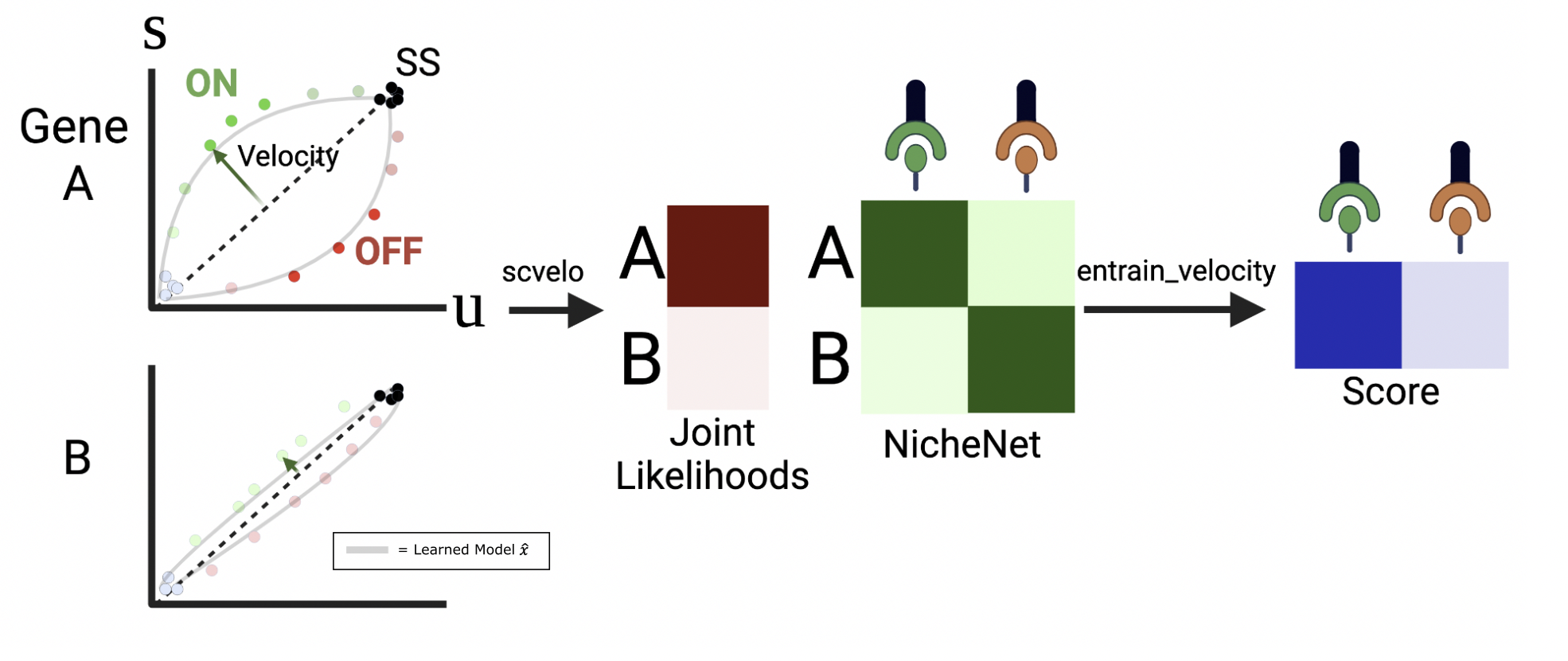


**Supplementary Figure S1:**For a given gene, scVelo fits a phase trajectory model to the un-spliced and spliced counts. Left, scatter plot: Cells are assigned to states on the phase diagram based on their distance to the phase model, with likelihoods of the assignment denoted by color opacity (black = steady-state ON, green = ON/upregulation, red = OFF/downregulation, light blue = steady-state OFF). Middle: Likelihoods across all cells are pooled into a joint likelihood for each gene (red squares). Right: ENTRAIN fits the NicheNet matrix to the likelihoods vector according to similarity between the velocity likelihoods and predicted ligand-gene relationships (green squares), resulting in a variable importance score (blue) for each ligand that denotes the significance of a ligand in driving the observed velocities.


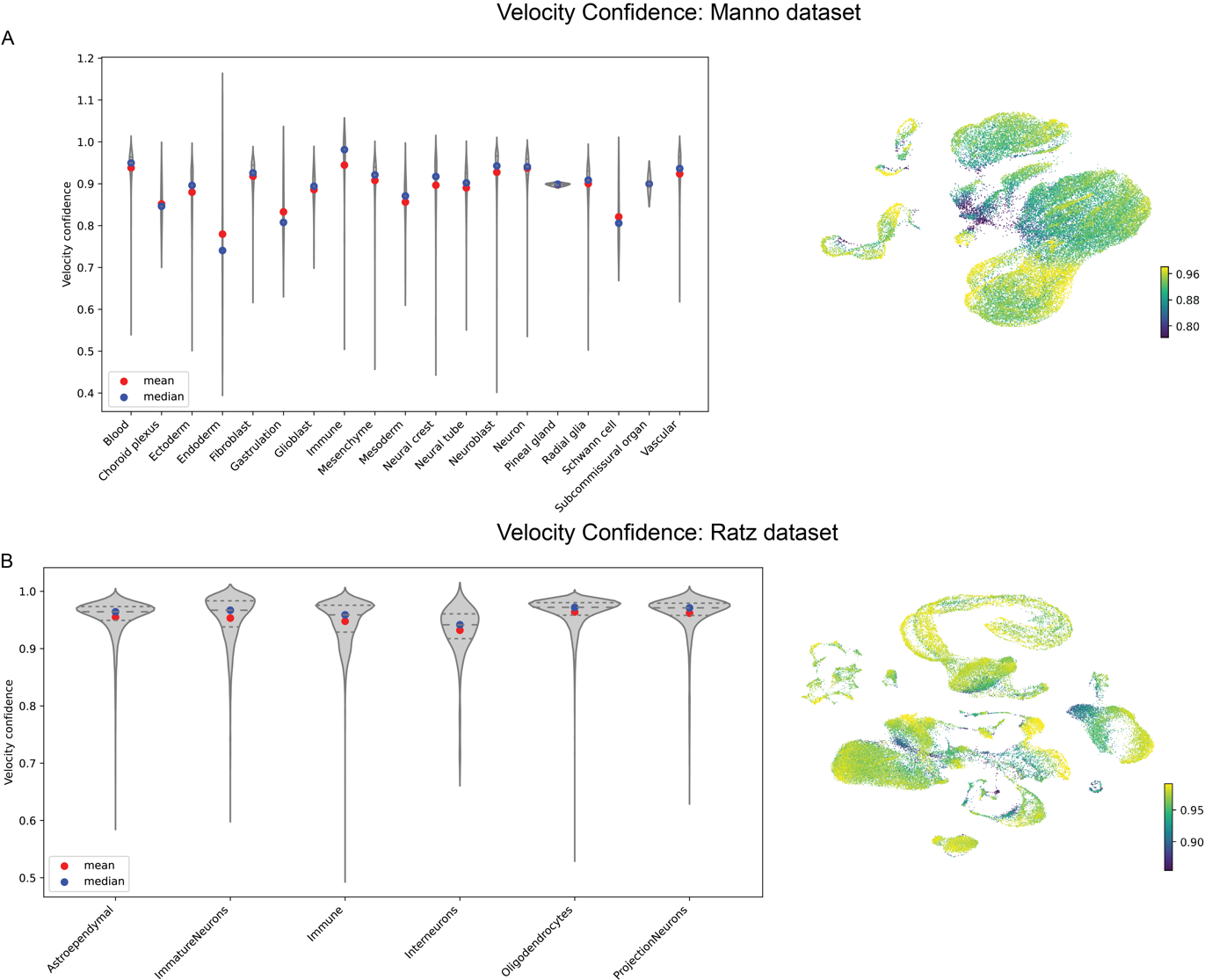


**Supplementary Figure S2:** Velocity confidence analysis of Manno (A) and Ratz (B) datasets per cell type cluster (left) and at single-cell (right) resolution.

**
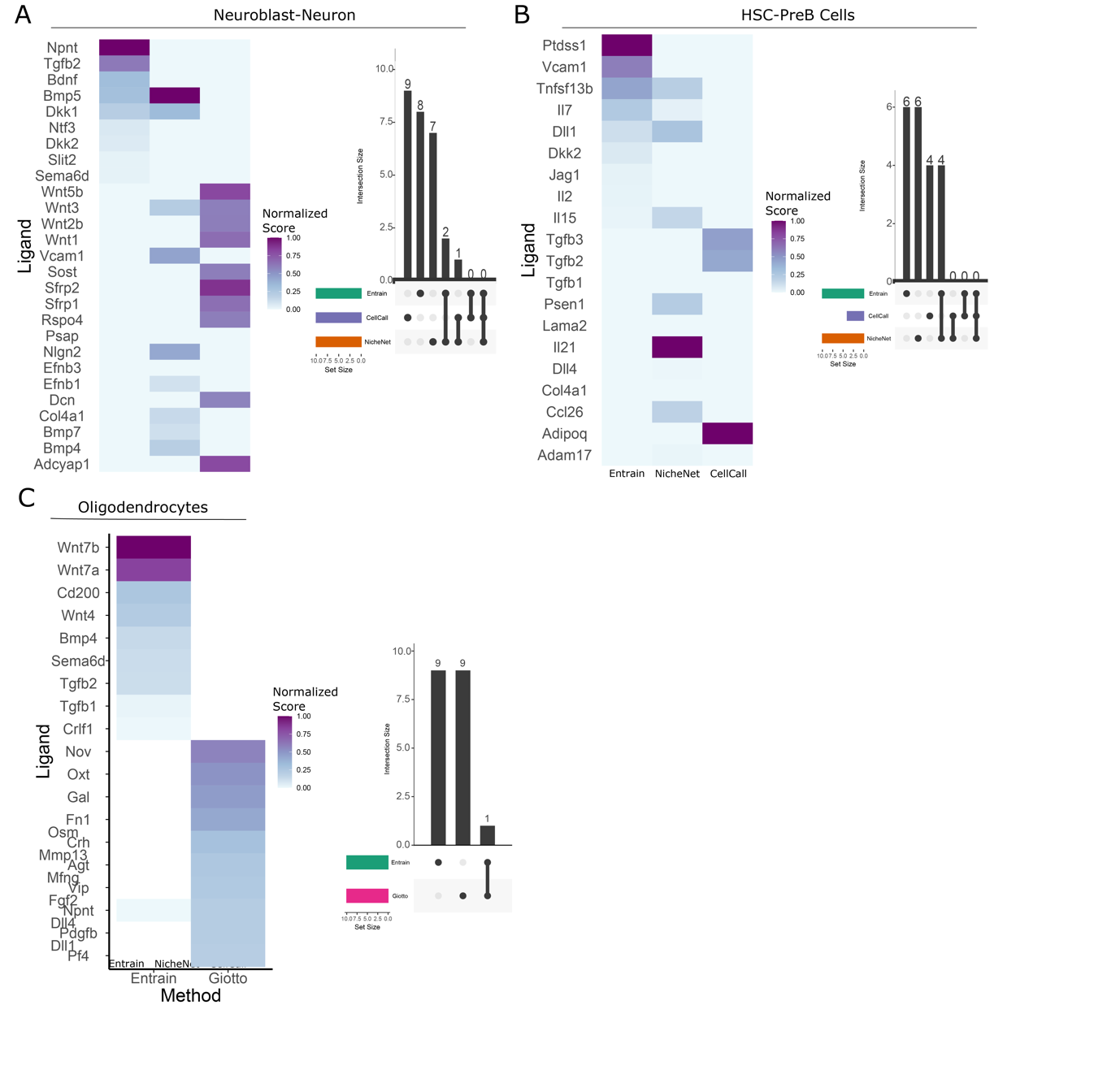
Supplementary Figure S3:**
(A) Left: Heatmap showing normalized scores of the top 10 ligands found by ENTRAIN, NicheNet and CellCall performed on the neuroblast-neuron subpopulation in EN. Right: UpsetR plot showing overlap between the ligands predicted by each method.

(B) As in (A) but for Progenitor-Pre-B Cell subpopulation in BME.

(C) Heatmap showing normalized scores of the top 10 ligands found by ENTRAIN and Giotto performed on the oligodendrocyte subpopulation of the Ratz et al. dataset


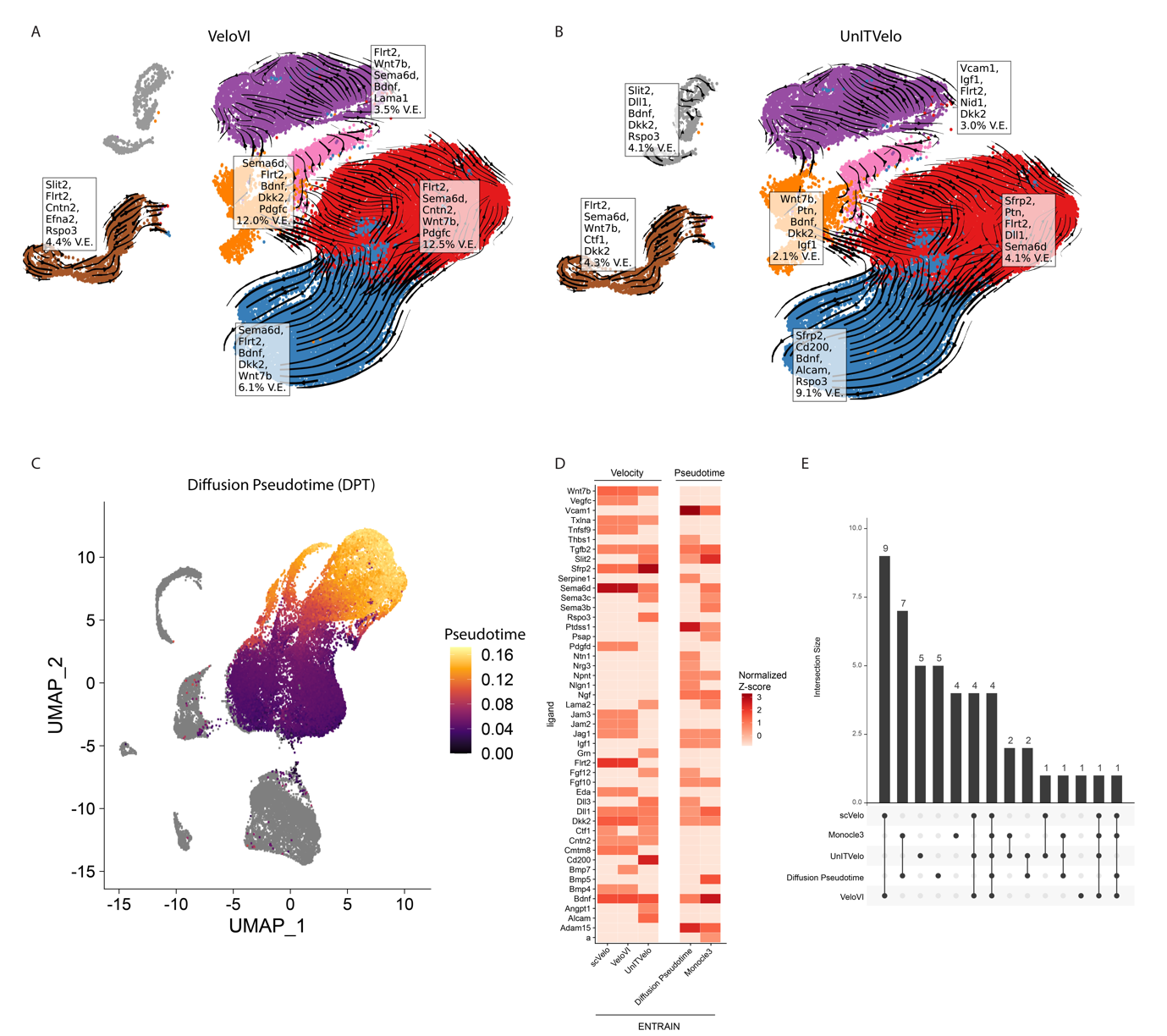


**Supplementary Figure S4:**

1. ENTRAIN-velocity results on velocity matrix recovered with VeloVI.
2. ENTRAIN-velocity results on velocity matrix recovered with UnITVelo.
3. Pseudotime values recovered with diffusion pseudotime.
4. Heatmap showing normalized scores of the top 20 ligands found by ENTRAIN-velocity. In order: ENTRAIN-velocity default (scVelo), VeloVI, UnITVelo, and Diffusion pseudotime, ENTRAIN-pseudotime default (Monocle 3).
5. UpsetR plot visualizing overlap between the top 20 ligands found by the five methods. Y axis indicates the number of ligands that are shared between the methods. Connecting lines show which methods have overlap. ENTRAIN Velocity - VeloVI overlap = 0.95; ENTRAIN pseudotime - Diffusion pseudotime overlap = 0.65; VeloVI - UnITVelo overlap = 0.45; ENTRAIN velocity - UnITVelo overlap = 0.5


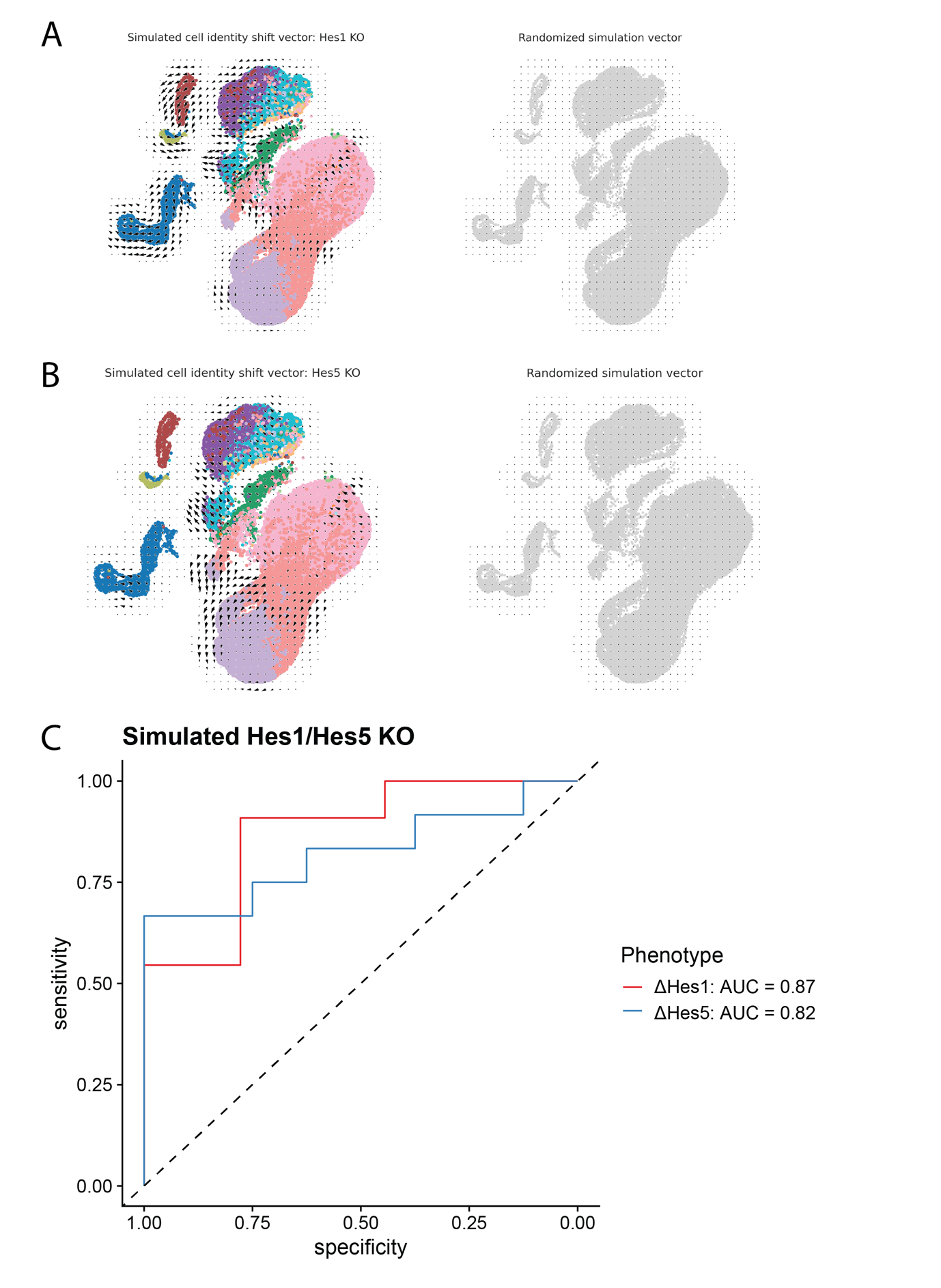

**Supplementary Figure S5:**

1. Left panel shows CellOracle simulated effect of gene expression shift vectors upon *in silico* knockout of *Hes1* on the Manno et al. dataset. Right panel shows randomized simulated gene expression shift vectors.
2. As in (A) but for *Hes5* knockout.
3. ROC curve showing literature support rates for ligands recovered by ENTRAIN when run on the simulated datasets.


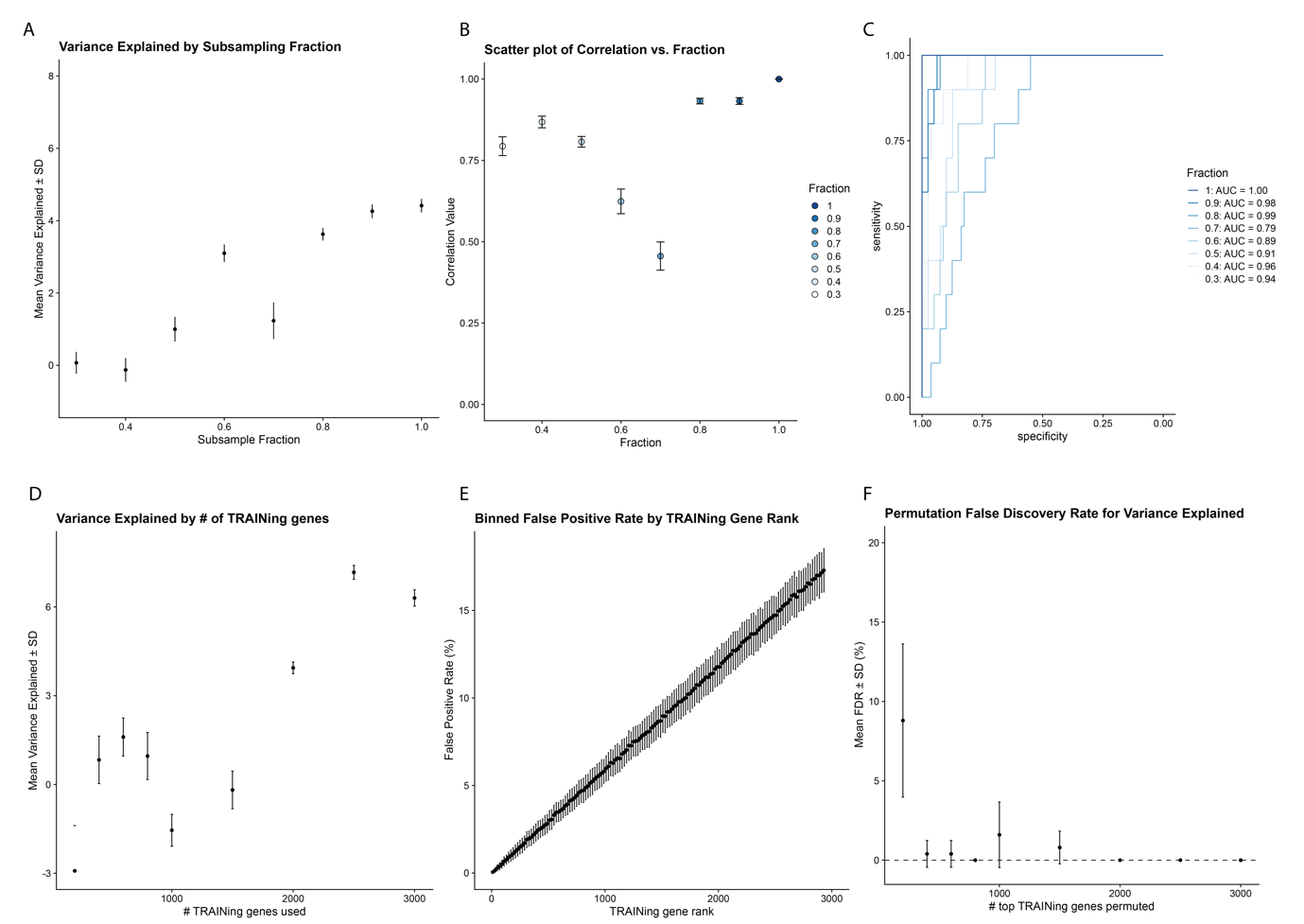


**Supplementary Figure S6:**

1. Variance Explained scores generated by ENTRAIN when run on subsampled fractions of Manno Neuron-neuroblast data. Each dot represents mean V.E. of n=5 repeated trials. Error bars represent standard deviation.
2. Spearman correlation between ENTRAIN ligand importance scores at various subsampling fractions compared to scores generated on non-subsampled data. Each dot represents mean correlation between subsampled versus non-subsampled ligand importance scores of n=5 subsampled datasets. Error bars represent standard deviation.
3. ROC curves of the top 10 ligands produced by ENTRAIN analysis on subsampled data, treating non-subsampled results as ground truth.
4. Variance Explained scores generated by ENTRAIN when n_top_genes (number of TRAINing genes used) is varied. Each dot represents mean V.E. of n=5 repeated trials. Error bars represent standard deviation.
5. Binned FDRs of TRAINing gene ranks upon randomly permuting gene names. A false discovery is counted when a permuted gene’s rank is higher than it’s non-permuted, original rank (x axis). Each dot represents mean of binned ranks (bin width = 20 ranks), error bars represent standard deviation.
6. FDRs of ENTRAIN variance explained scores. For each value of n_top_genes (x axis), the corresponding TRAINing genes were randomly permuted and likelihoods used as input for ENTRAIN analysis. A false discovery is counted when the V.E. resulting from permuted input exceeds the V.E. from the non-permuted baseline. Each dot represents mean of 10 FDR’s, each FDR resulting from 50 tests. Error bars represent standard deviation. Dashed line represents 0.

**References**

1. Browaeys, R., Saelens, W. & Saeys, Y. NicheNet: modeling intercellular communication by linking ligands to target genes. *Nature Methods* **17**, 159-162 (2020).

2. Page, L., Brin, S., Motwani, R. & Winograd, T. in The Web Conference (1999).

3. Kamimoto, K. et al. Dissecting cell identity via network inference and in silico gene perturbation. *Nature* **614**, 742-751 (2023).

4. Stuart, T. et al. Comprehensive Integration of Single-Cell Data. *Cell* **177**, 1888-1902.e1821 (2019).

5. Blondel, V.D., Guillaume, J.-L., Lambiotte, R. & Lefebvre, E. Fast unfolding of communities in large networks. *Journal of statistical mechanics: theory and experiment* **2008**, P10008 (2008).

6. Hao, Y. et al. Integrated analysis of multimodal single-cell data. *Cell* **184**, 3573-3587.e3529 (2021).

7. McInnes, L., Healy, J. & Melville, J. Umap: Uniform manifold approximation and projection for dimension reduction. *arXiv preprint arXiv:1802.03426* (2018).
