# Supplementary figures and images for: ENTRAIN: integrating trajectory inference and gene regulatory networks with spatial data to co-localize the receptor–ligand interactions that specify cell fate
Source: Bioinformatics. 2023 Dec 19;39(12):btad765. doi: 10.1093/bioinformatics/btad765 (PMC10752580; doi:10.1093/bioinformatics/btad765)

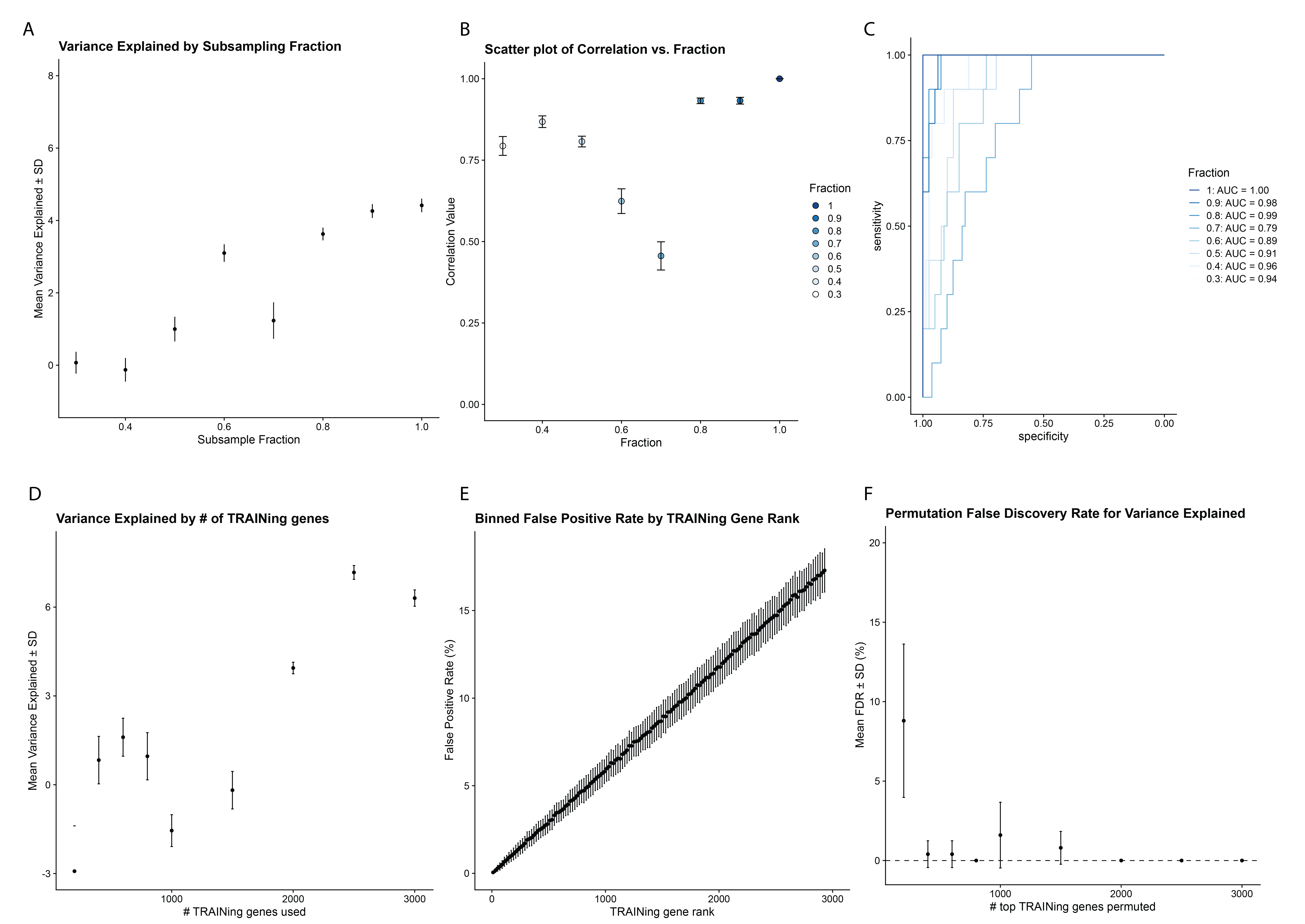

Supplement: btad765_Supplementary_Data [file btad765_supplementary_data.zip › supp6_validation_dpi.png]

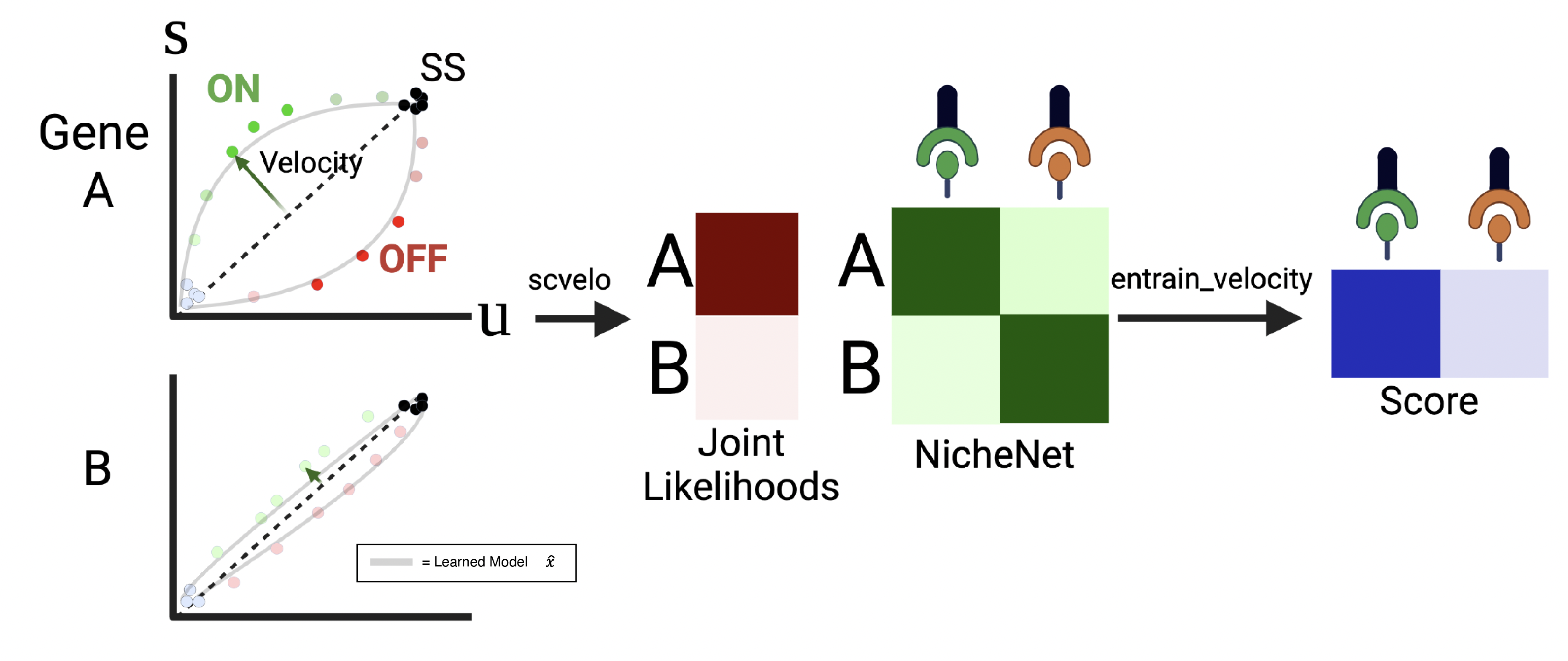

Supplement: btad765_Supplementary_Data [file btad765_supplementary_data.zip › Supp1_Velo_Method_dpi.png]

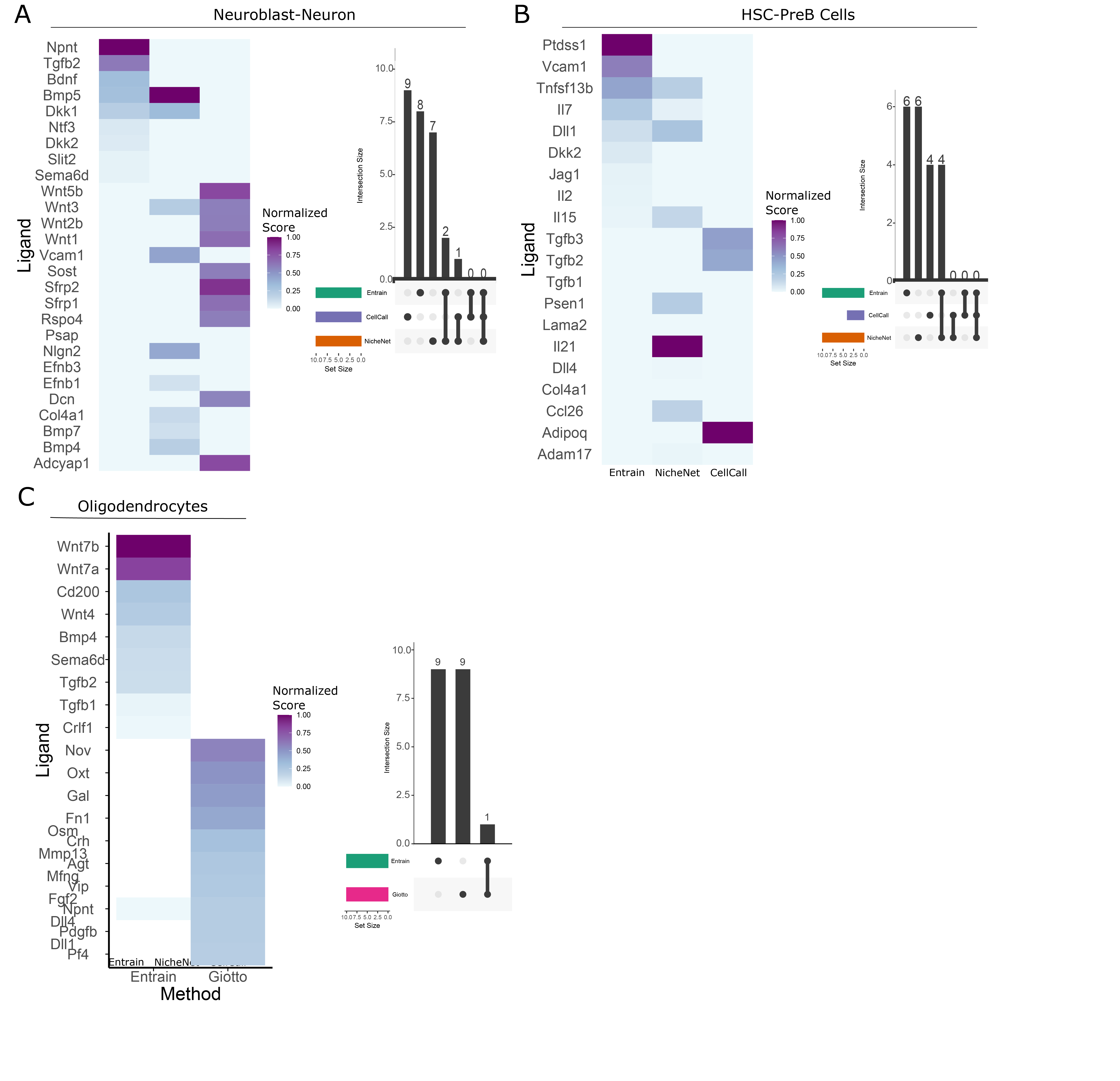

Supplement: btad765_Supplementary_Data [file btad765_supplementary_data.zip › supFig3_comparison_dpi.png]

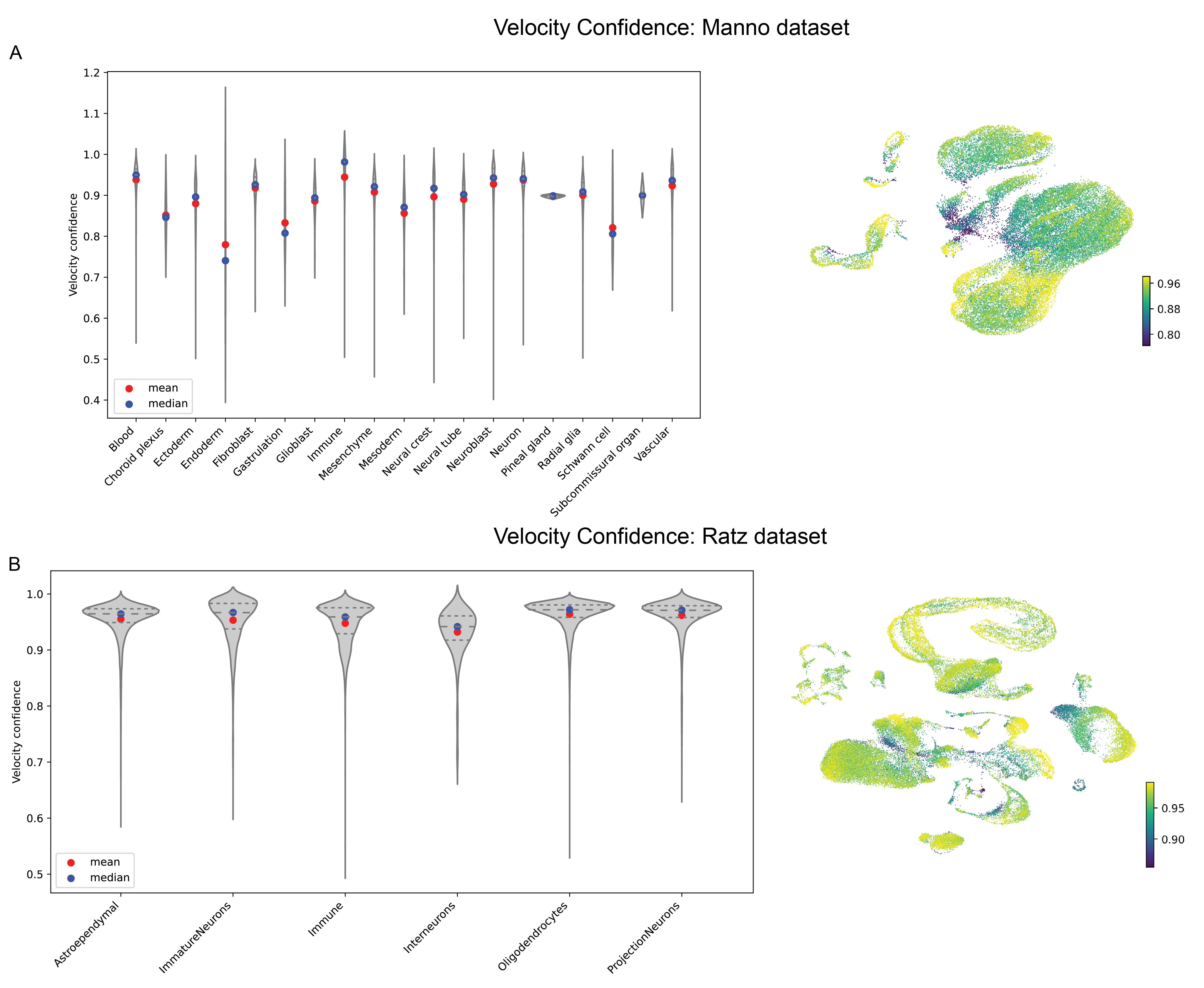

Supplement: btad765_Supplementary_Data [file btad765_supplementary_data.zip › supp_veloconfidence_dpi.png]

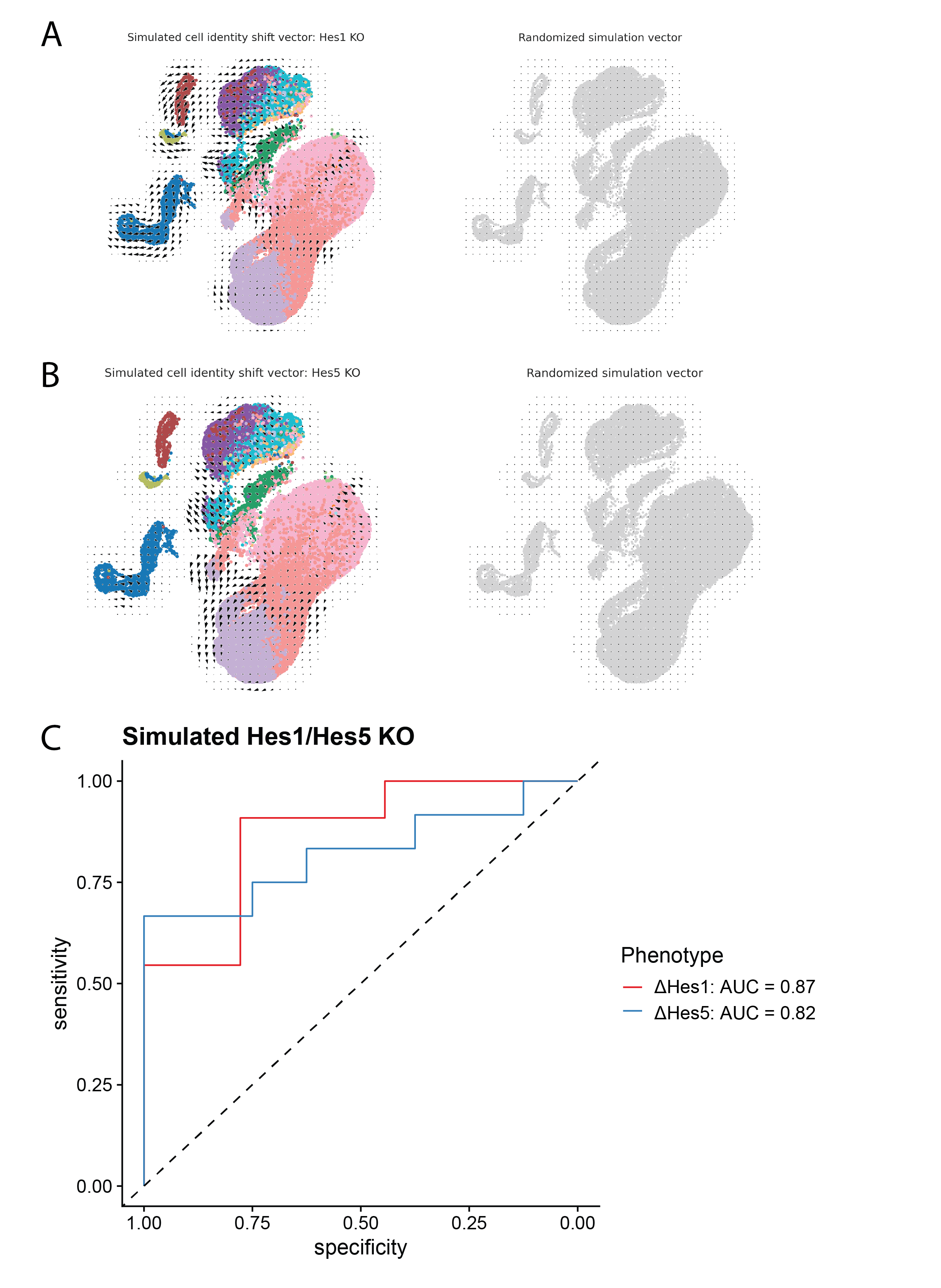

Supplement: btad765_Supplementary_Data [file btad765_supplementary_data.zip › supp5_celloracle_dpi.png]
